# Effects of Ranolazine on Vascular Adrenergic Receptors in Rabbit Aorta

**DOI:** 10.7150/ijms.128068

**Published:** 2026-03-17

**Authors:** Adrian Jorda, Maria Dolores Mauricio, Solanye Guerra-Ojeda, Jose M. Vila, Soraya L. Valles, Martin Aldasoro

**Affiliations:** 1 School of Medicina, University of Valencia, Spain.; 2 School of Nursing and Podiatry, University of Valencia, Spain.

**Keywords:** Ranolazine, adrenergic α_1_, α_2_, β_2_, β_3_ receptors, adrenergic receptor expression, vasoconstriction, vasodilatation.

## Abstract

**Background:**

Different mechanisms of action have been proposed for Ranolazine (Rn), mainly the inhibition of the late sodium current and antagonism of α₁-adrenergic receptors. In the present study, we evaluated the possible involvement of other adrenergic receptors, specifically α₂, β₂, and β₃, as mediators of the vascular effects of Rn.

**Methods:**

Segments of rabbit aorta were mounted in an organ bath. Electrical field stimulation (EFS; 2, 4, and 8 Hz) induced frequency-dependent contractions that were abolished by tetrodotoxin, prazosin, or guanethidine (10⁻⁶ M), confirming the neural origin of the vascular responses. The effects of Rn on vascular responses to adrenergic stimulation were evaluated by incubating the preparations with increasing concentrations of the drug (10⁻⁷-10⁻⁴ M) for 20 minutes prior to neural stimulation (4 Hz). The involvement of α₁-, α₂-, β₂-, or β₃-adrenergic receptors was assessed using specific antagonists (10⁻⁶ M): prazosin (α₁), yohimbine (α₂), butaxamine (β₂), and SR59230A (β₃). Subsequently, the sequence of electrical field stimulations was performed in the presence of Rn. Expression levels of α₁-, α₂-, β₂-, and β₃-adrenergic receptors were determined by Western blot analysis.

**Results:**

Rn decreases the contractile effect induced by adrenergic nerve stimulation in the rabbit aorta. In the presence of prazosin or yohimbine, the vasoconstrictor response was significantly reduced. However, incubation with butaxamine or SR59230A significantly increased the contractile response to adrenergic nerve stimulation. The protein expression of α_1_ and α_2_ receptors significantly decreased compared to the control when incubated with Rn. In contrast, the expression of β_2_ and β_3_ receptors increased only at 10⁻⁷ M, a concentration lower than that reached with therapeutic doses of Rn.

**Conclusion:**

Rn inhibits the vasoconstrictor response to adrenergic nerve stimulation through an antagonistic effect on α_1_ and α_2_ receptors and enhancing the vasodilatory responses mediated by β_2_ and β_3_ adrenergic receptors.

## Introduction

Ranolazine [(+)-N-(2,6-dimethylphenyl)-4(2-hydroxy-3-(2-methoxyphenoxy)-propyl)-1-piperazine acetamide] (Rn) is a piperazine-derived drug approved by the FDA in 2006, whose clinical indications are mainly focused on the treatment of chronic coronary ischemia refractory to other therapies 1, 2. However, in recent years, the medical indications for Rn have expanded, including its use in the treatment of cardiac arrhythmias such as atrial fibrillation, and in coronary endothelial dysfunction, by increasing the release of various vasodilator factors such as nitric oxide and prostanoids. Moreover, it has also been shown to reduce oxidative stress by inhibiting fatty acid oxidation and promoting greater glucose oxidation 3.

Different metabolic effects have also been described, including a reduction in blood glucose and glycated hemoglobin (hemoglobin A1C, HbA1c) levels 4, 5, as well as improvement in insulin secretion and β-cell survival in diabetic mice 6. Moreover, evidence suggests that Rn may improve various cognitive processes in type II diabetes 7, 8. In our laboratory, we have demonstrated that Rn enhances tissular insulin sensitivity both in the vascular wall and in cells of the central nervous system 9, 10.

Other effects of Rn target cells in the central nervous system by reducing neuronal excitability, thus acting as an anticonvulsant agent 11, 12. In addition, it has been proposed as a potential treatment for neuropathic pain 13. There is also evidence of a protective effect against the development of different types of dementia, particularly those related to oxidative and inflammatory mechanisms 14, 9, 15.

Although the mechanism of action of Rn is not exactly known, it has been shown that, at therapeutic concentrations, it selectively inhibits the late inward Na⁺ current (I_NaL), thus reducing the intracellular concentration of Na⁺, which would inhibit the activity of the Na⁺-Ca²⁺ exchanger and the subsequent entry of Ca²⁺ into the cells (Ca²⁺_i). In this way, intracellular ionic homeostasis would be preserved, thereby reducing the tension of both ventricular and vascular muscle fibers 16, 17. Rn also acts on other cellular ion channels such as TASK-1 potassium channels, whose inhibition would contribute to its antiarrhythmic effects 18.

Other mechanisms of action have also been described for Rn, among which the antagonistic effect it exerts on α₁-adrenergic receptors stands out. In fact, in our laboratory we have demonstrated an antagonism with α₁ adrenergic receptors in the saphenous vein used in coronary bypass surgery 19. Nevertheless, it is possible that Rn also interacts with other adrenergic receptors, particularly those of the beta type. The aim of this study was to evaluate the possible interactions between Rn and the β₂-, β₃-, α₁-, and α₂-adrenergic receptors.

## Materials and Methods

### Animal Model

The investigation was carried out in accordance with the ethical standards in animal experimentation established by EU Directive 2010/63 and Spanish Royal Decree (RD) 1201/2005. The experiments were carried out using tissue samples according to procedure 2017/VSC/PEA/00049 type 2, authorized by the Bioethics Committee of the University of Valencia, Spain. Forty-two male New Zealand white rabbits, weighing 3.2-3.8 kg, were used in this study and housed in a 12:12 h light/dark cycle at a constant room temperature of 22 ºC and 60% humidity. Animals were euthanized following heparinization and anesthesia (sodium thiopental 60 mg/kg i.v.).

### Preparation of Vascular Rings

Organ bath experiments were carried out as previously described 20. Abdominal aorta was isolated and cut into 4-mm rings for isometric recording of tension. Two stainless steel L-shaped pins were introduced through the lumen of the vascular rings. One pin was fixed to the wall of the organ bath, and the other one was connected to a force-displacement transducer (FT03; Grass Instruments, West Warwick, RI, USA). Variations in isometric force were registered on a Macintosh computer (Apple Corp., Cupertino, CA, USA) using the Chart, version 7, and a MacLab/8e data acquisition system (AD Instruments). Individual rings were suspended in a 5 mL bath with a modified Krebs-Henseleit solution containing (mM) NaCl, 115; CaCl_2_, 2.5; KCl, 4.6; MgCl_2_.6H_2_O, 1.2; NaHCO_3_, 25; glucose, 11.1; and disodium EDTA, 0.01, with 95% O_2_ and 5% CO_2_ to obtain a pH 7.3-7.4, and temperature was held at 37 ºC. The optimal resting tension for vascular rings was 3.5 g, and aortic preparations were allowed to equilibrate for 3 h. The contractile capacity of vascular smooth muscle was evaluated by the maximum response to KCl (60 mM). The endothelium was considered functional if relaxation to acetylcholine (10^-6^ M), in aortic rings precontracted with noradrenaline, was ≥ 70%. Vascular rings with dysfunctional endothelium in the control conditions were excluded.

## Experimental Procedure

### Periarterial Adrenergic Nerve Stimulation

To obtain adrenergic nerve stimuli, electrical field stimulation (EFS) was applied through two platinum electrodes placed on both sides of the aortic segments with a 5-mm separation between the two electrodes. The electrodes were connected to a multichannel stimulator (Grass S88). The correspondence between frequency and vasomotor response was studied in a determinate range of frequencies, specifically 2 and 4 Hz, with the application of 25 V stimuli (supramaximal voltage) of 0.25 ms duration for each pulse for 30 s of total duration of stimulation. The evaluation of the neurogenic nature of the contractile response to EFS was carried out by incubating the vascular segments for 15 min with tetrodotoxin (TTX) (10^-6^ M), a blocker of voltage dependent Na^+^ channels and, therefore, an inhibitor of the nerve conduction of the neural fibers present in the vascular wall; guanethidine (10^-6^ M), a blocker of the release of noradrenaline, neurotransmitter of the adrenergic nervous axons; therefore, it also inhibits adrenergic neurotransmission; or with an α_1_ adrenergic postsynaptic receptor antagonist, prazosin (10^-6^ M). Different stimulation rounds (2 and 4 Hz) were provoked, with 5 min intervals between each stimulus of increasing frequency. These series of stimuli were applied again 10 min after adding TTX (10^-6^ M), guanethidine (10^-6^ M), or prazosin (10^-6^ M), to the organ bath. As a control group, in another series of vascular segments, electrical field stimuli were performed without the presence of adrenergic nervous system blockers.

### Interaction between Rn with the nervous adrenergic system

The possible interaction of Rn with the sympathetic-adrenergic nervous system was assessed by incubating the vascular rings with Rn (10^-7^-10^-4^ M) for fifteen minutes prior to the application of adrenergic nervous stimuli at frequency of 4 Hz.

### Involvement of α₁-adrenergic receptors in the vascular responses to Rn

The involvement of α₁-adrenergic receptors in the responses of the abdominal aorta to Rn after stimulation of the adrenergic nervous system was evaluated by incubating the vascular segments with Prazosin (10⁻⁶ M) for fifteen minutes prior to the application of EFS (4 Hz) and Rn (10⁻⁷-10⁻⁴ M).

### Involvement of α₂-adrenergic receptors in the vascular responses to Rn

The involvement of α₂-adrenergic receptors in the responses of the abdominal aorta to Rn after stimulation of the adrenergic nervous system was evaluated by incubating the vascular segments with Yohimbine (10⁻⁶ M) for fifteen minutes prior to the application of EFS (4 Hz) and Rn (10⁻⁷-10⁻⁴ M).

### Involvement of β₂-adrenergic receptors in the vascular responses to Rn

The involvement of β₂-adrenergic receptors in the responses of the abdominal aorta to Rn after stimulation of the adrenergic nervous system was evaluated by incubating the vascular segments with Butaxamine (10⁻⁶ M) for fifteen minutes prior to the application of EFS (4 Hz) and Rn (10⁻⁷-10⁻⁴ M).

### Involvement of β₃-adrenergic receptors in the vascular responses to Rn

The involvement of β₃-adrenergic receptors in the responses of the abdominal aorta to Rn after stimulation of the adrenergic nervous system was evaluated by incubating the vascular segments with SR59230A (10⁻⁶ M) for fifteen minutes prior to the application of EFS (4 Hz) and Rn (10⁻⁷-10⁻⁴ M).

### Drugs

The drugs used were Potassium Chloride (KCl, Merck, Darmstadt, Germany), Insulin, Acetylcholine, Noradrenaline Hydrochloride, Prazosin, Tetrodotoxin, Guanethidine, Yohimbine, Butaxamine, SR59230A and Ranolazine [(+)-N-(2,6-dimetilfenil)-4(2-hidroxi- 3-(2-metoxifenoxi)-propil)-1-piperazina acetamida], (3,4-dihydro-4-(2-pyrimidinylmethyl)-7-[4-(trifluoromethoxy)phenyl]-1,4benzoxazepin-5(2H)-one) (Sigma-Aldrich, Madrid, Spain). Concentrated drug solutions were obtained with bi-distilled water, except for prazosin, which were dissolved in ethanol.

### Western-blot analysis

Protein extracts from vascular rings of abdominal aorta were mixed with an equal volume of SDS buffer (0.125 M Tris-HCl, pH 6.8, 2% SDS, 0.5% (v/v) 2-mercaptoethanol, 1% bromophenol blue, and 19% glycerol) and then heated for 5 minutes. Protein concentration was measured using a modified Lowry method. Proteins were separated via SDS-PAGE and transferred to nitrocellulose membranes using standard techniques. Membranes were blocked with 5% dried milk in TBS containing 0.05% Tween-20, then incubated with the appropriate antibodies according to the manufacturer's instructions. The blots were washed three times for 15 minutes each with washing buffer (phosphate-buffered saline, 0.2% Tween-20), followed by a 1-hour incubation with a secondary horseradish peroxidase-linked anti-rabbit or anti-mouse IgG antibody (Cell Signaling Technologies, Barcelona, Spain). Afterward, the blots were washed three times and developed using the enhanced chemiluminescence (ECL) method as per the manufacturer's instructions (Pharmacia Biotech, San Francisco, CA, USA). Autoradiographic signals were quantified with a Bio-Rad scanning densitometer. The following antibodies were used: Anti-α_1_ Adrenoreceptor (ab137123), Anti-α_2_ Adrenoreceptor (ab85570), Anti-β_2_ Adrenoreceptor (ab176490), Anti-β_3_ Adrenoreceptor (ab94506) and anti-tubulin (ab6046) (Abcam biotechnology).

### Statistical analysis

Data are expressed as means ± SEM; *n* indicates the number of rabbits. At least eight artery rings were obtained from each case. The results were evaluated statistically by means of paired or unpaired Student's *t* test or one-way analysis of variance. The probability value of < 0.05 was significant.

## Results

### Effects of Electrical Field Stimulation (EFS)

**Figure 1:**
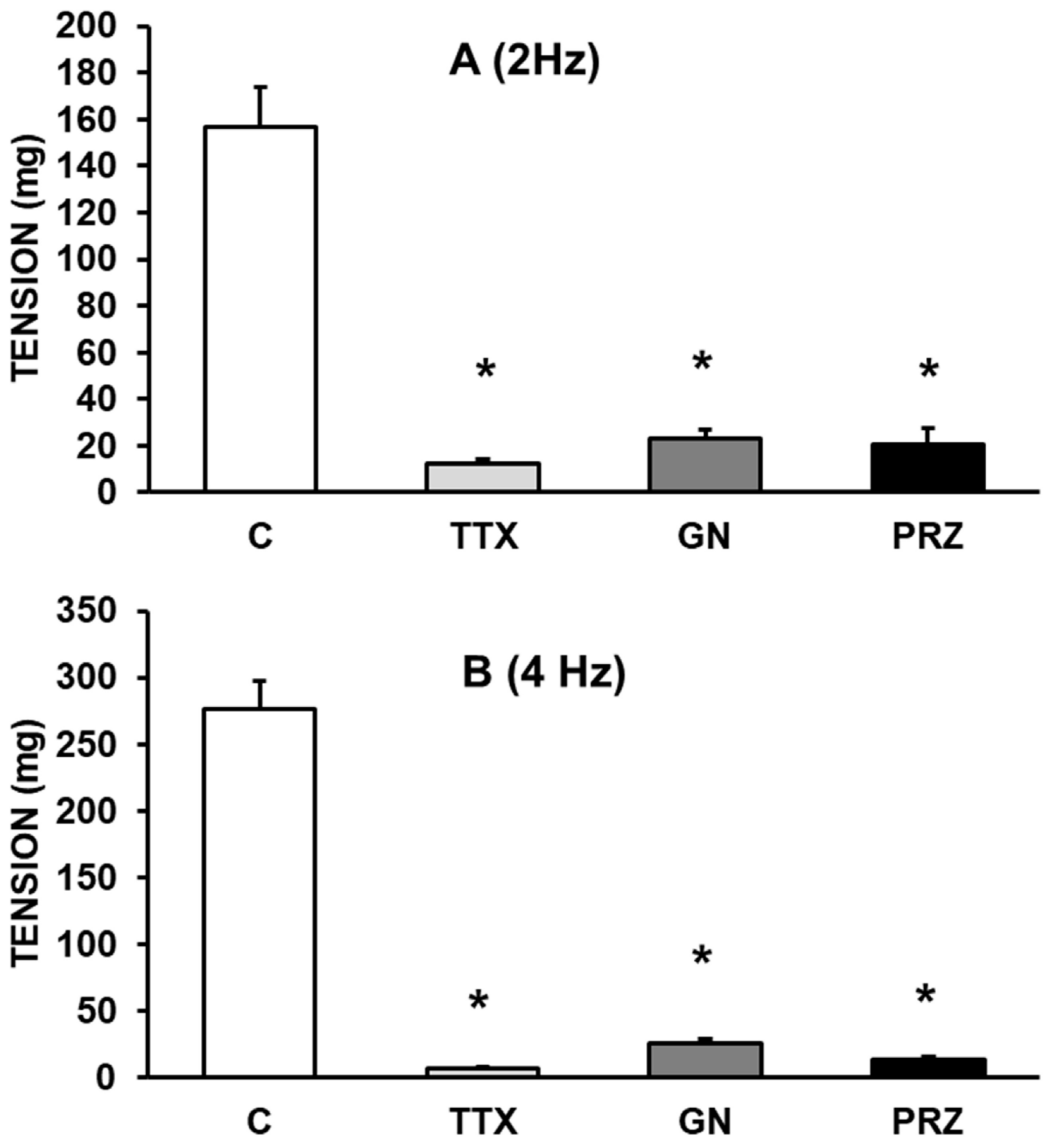
Effects of EFS at 2 Hz **(A)** and 4 Hz **(B)** on rabbit aorta in the absence (control, n = 8) and in the presence of tetrodotoxin (TTX) (10^-6^ M, n = 7), guanethidine (GN) (10^-6^ M, n = 6), or prazosin (PRZ) (10^-4^ M, n = 6). Values are means ± SEM shown by vertical bars. *p < 0.05 *vs.* control.

EFS (at 2 and 4 Hz) elicited frequency-dependent contractions of rabbit aortic rings at resting tension. The vasoconstriction induced by EFS was blocked after incubating the aortic segments with TTX (10^-6^ M), guanethidine (10^-6^ M), or prazosin (10^-6^ M). Taken together, these results indicate that the vasoconstrictor effect induced by EFS is mediated by adrenergic fibers that release norepinephrine from nerve axons following its binding to α_1_-adrenergic receptors (Figure 1, A and B).

### Effects of Ranolazine on the responses of the aortic segments to sympathetic nerve stimulation

**Figure 2:**
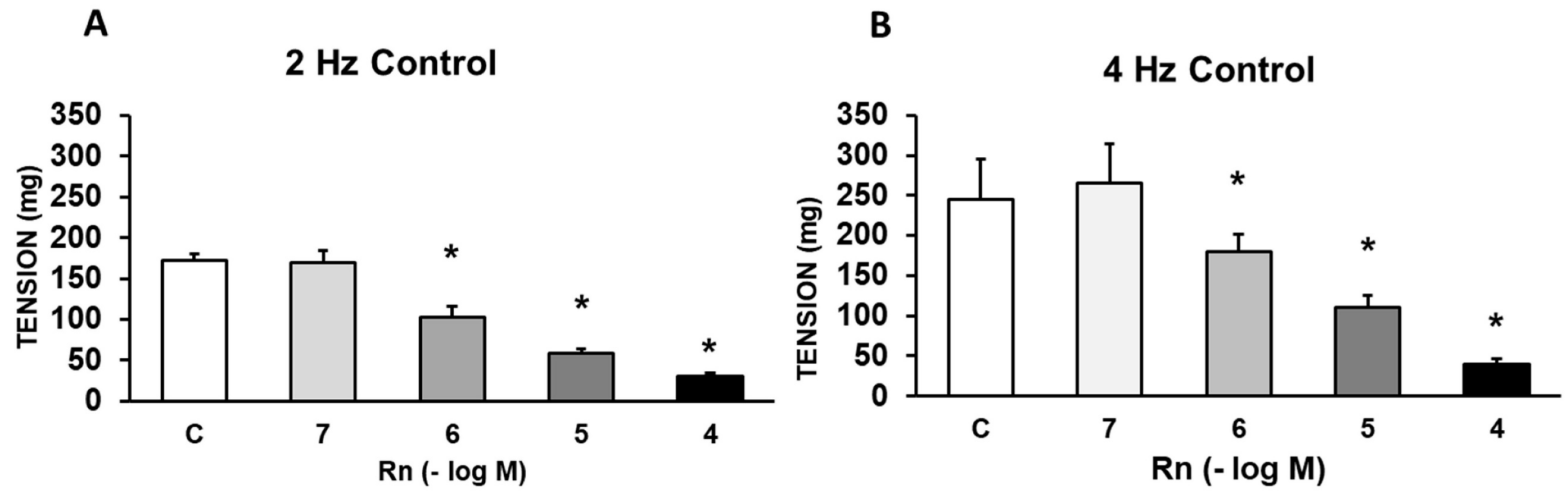
Effects of EFS at 2 Hz **(A)** and 4 Hz **(B)** on rabbit aorta in the absence (control, n = 6) and in the presence of Rn (10^-7^-10^-4^ M, n = 7). Values are means ± SEM shown by vertical bars. *p < 0.05 *vs.* control.

Rn (10^-6^ - 10^-4^ M) caused significant concentration-dependent decrease in the vasoconstrictor response induced by adrenergic nerve stimulation (4 Hz). Lower concentrations of Rn (10^-7^) do not cause any vascular effect (Figure 2).

### Interactions between Ranolazine and α1 adrenergic receptors

**Figure 3:**
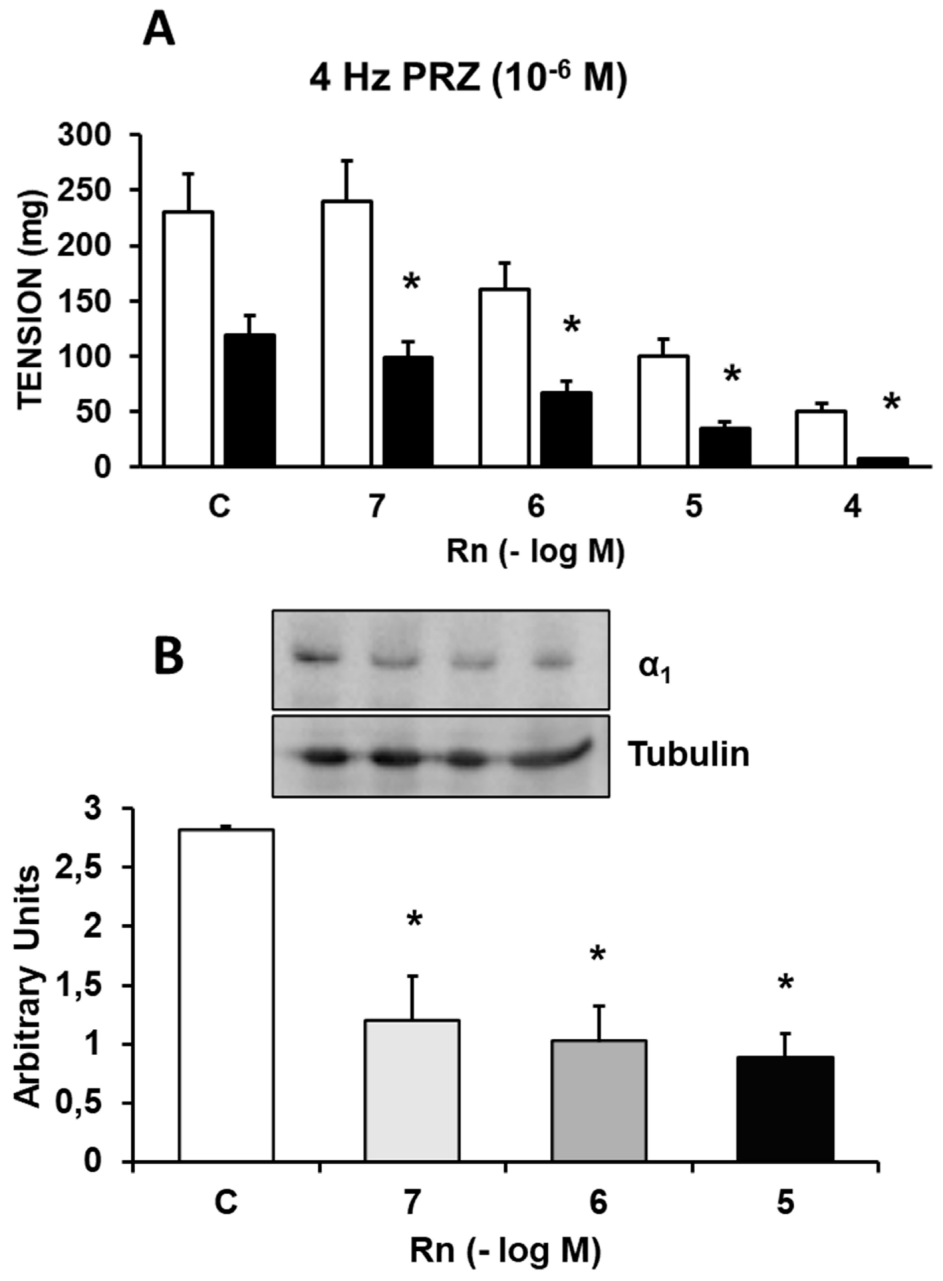
**(A)** Contractile effects of EFS at 4 Hz on rabbit aorta in the absence (control, n = 7) and in the presence of Rn (10^-7^-10^-4^ M) and without PRZ (□) (n = 7) or previously incubated with PRZ (■) (10^-6^ M, n = 6). Values are means ± SEM shown by vertical bars. *p < 0.05 *vs.* control with PRZ. **(B)** Protein expression of α_1_ adrenergic receptors by Western-blot. A representative immunoblot is shown in each panel. Data are the mean ± SD of four independent experiments. *p < 0.05 vs. control.

In the presence of Prazosin (10⁻⁶ M), an α₁-adrenergic receptor antagonist, the contractile response induced by EFS was reduced by 48-56%. Rn (10⁻⁷ - 10⁻⁴ M) inhibited the contractile responses to EFS by a percentage similar to that observed in experiments without Prazosin (Figure 3A). Furthermore, Rn, at all concentrations tested, decreased the protein expression of α₁-adrenergic receptors (Figure 3B).

Together, these results indicate that Rn continues to inhibit the contractile response to EFS, which was previously partially blocked by Prazosin (10⁻⁶ M). Given that Prazosin at a concentration of 10⁻⁴ M completely inhibits the contractile response to EFS, it is reasonable to assume that the relaxation induced by Rn under these conditions is due to α₁-adrenergic antagonism, consistent with the reduced expression of α₁ receptors in the presence of Rn.

### Interactions between Ranolazine and α_2_ adrenergic receptors

**Figure 4:**
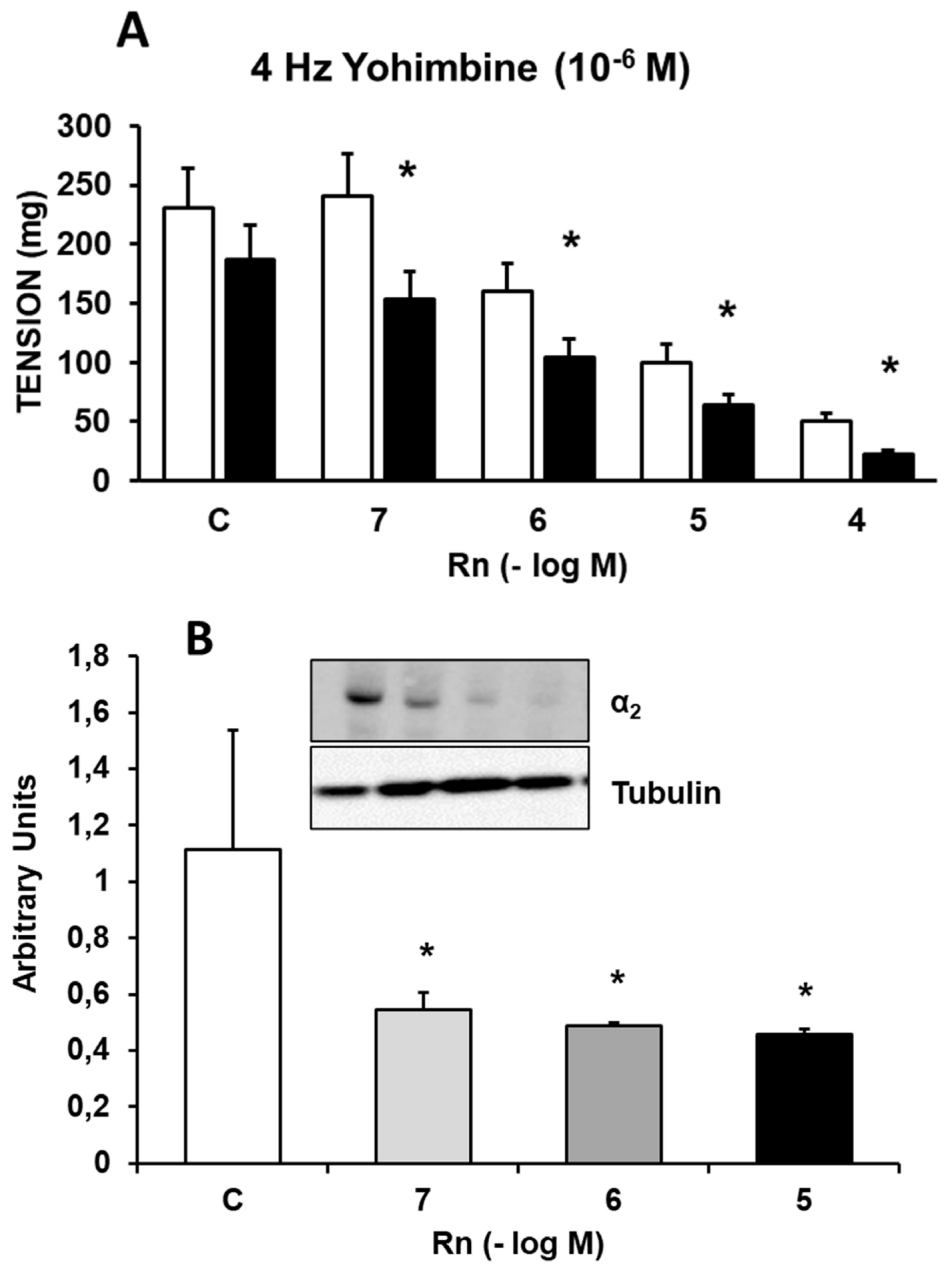
** (A)** Contractile effects of EFS at 4 Hz on rabbit aorta in the absence (control, n = 8) and in the presence of Rn (10^-7^-10^-4^ M) and without Yohimbine (□) (n = 8) or previously incubated with Yohimbine (■) (10^-6^ M, n = 7). Values are means ± SEM shown by vertical bars. *p < 0.05 vs. control with Yohimbine. **(B)** Protein expression of α_2_ adrenergic receptors by Western-blot. A representative immunoblot is shown in each panel. Data are the mean ± SD of four independent experiments. *p < 0.05 vs. control.

Incubation with Yohimbine (10⁻⁶ M), an α₂-adrenergic receptor antagonist, decreased the contractile response induced by EFS by 23-31%. Rn (10⁻⁷ - 10⁻⁴ M) continued to inhibit the contractile responses to EFS by a percentage similar to that observed in experiments without Yohimbine (Figure 4A). Additionally, Rn, at all concentrations tested, decreased the protein expression of α₂-adrenergic receptors (Figure 4B).

Together, these results indicate that Rn continues to inhibit the contractile response to EFS, which was previously partially blocked by Yohimbine (10⁻⁶ M). Overall, these findings suggest that Rn also acts as an antagonist of α₂-adrenergic receptors.

### Interactions between Ranolazine and β_2_ adrenergic receptors

**Figure 5:**
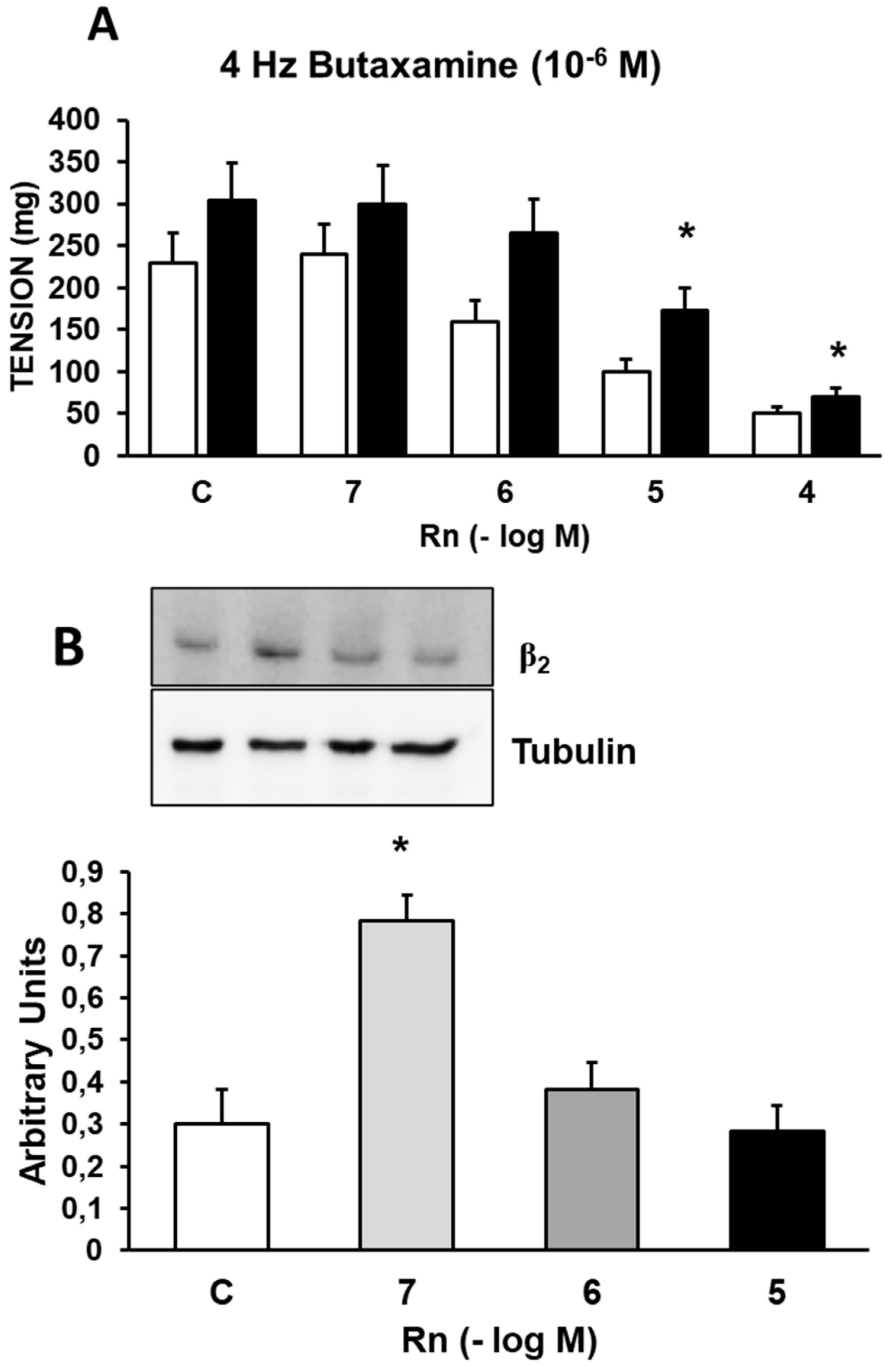
**(A)** Contractile effects of EFS at 4 Hz on rabbit aorta in the absence (control, n = 7) and in the presence of Rn (10^-7^-10^-4^ M) and without Butaxamine (□) (n = 8) or previously incubated with Butaxamine (■) (10^-6^ M, n = 8). Values are means ± SEM shown by vertical bars. *p < 0.05 *vs*. control with Butaxamine. **(B)** Protein expression of β2 adrenergic receptors by Western-blot. A representative immunoblot is shown in each panel. Data are the mean ± SD of five independent experiments. *p < 0.05 *vs.* control. # < 0,05* vs*. control sin Butaxamine.

Incubation with Butaxamine (10⁻⁶ M), a β₂-adrenergic receptor antagonist, increased the contractile response induced by EFS by 21% (Figure 5A) compared to control values (Figure 2A). Rn (10⁻⁷ and 10⁻⁶ M) did not induce changes in the contractile response to EFS. On the other hand, Rn (10⁻⁵ and 10⁻⁴ M) reduced the contractile responses to EFS (Figure 5A), but to a lesser extent than in aortic segments not incubated with Butaxamine (Figure 2A). Additionally, Rn induced a very significant increase in β₂ receptor expression only at the concentration of 10⁻⁷ M (Figure 5B). These data suggest that β₂ receptors may be involved in the relaxing responses elicited by Rn.

### Interactions between Ranolazine and β_3_ adrenergic receptors

**Figure 6:**
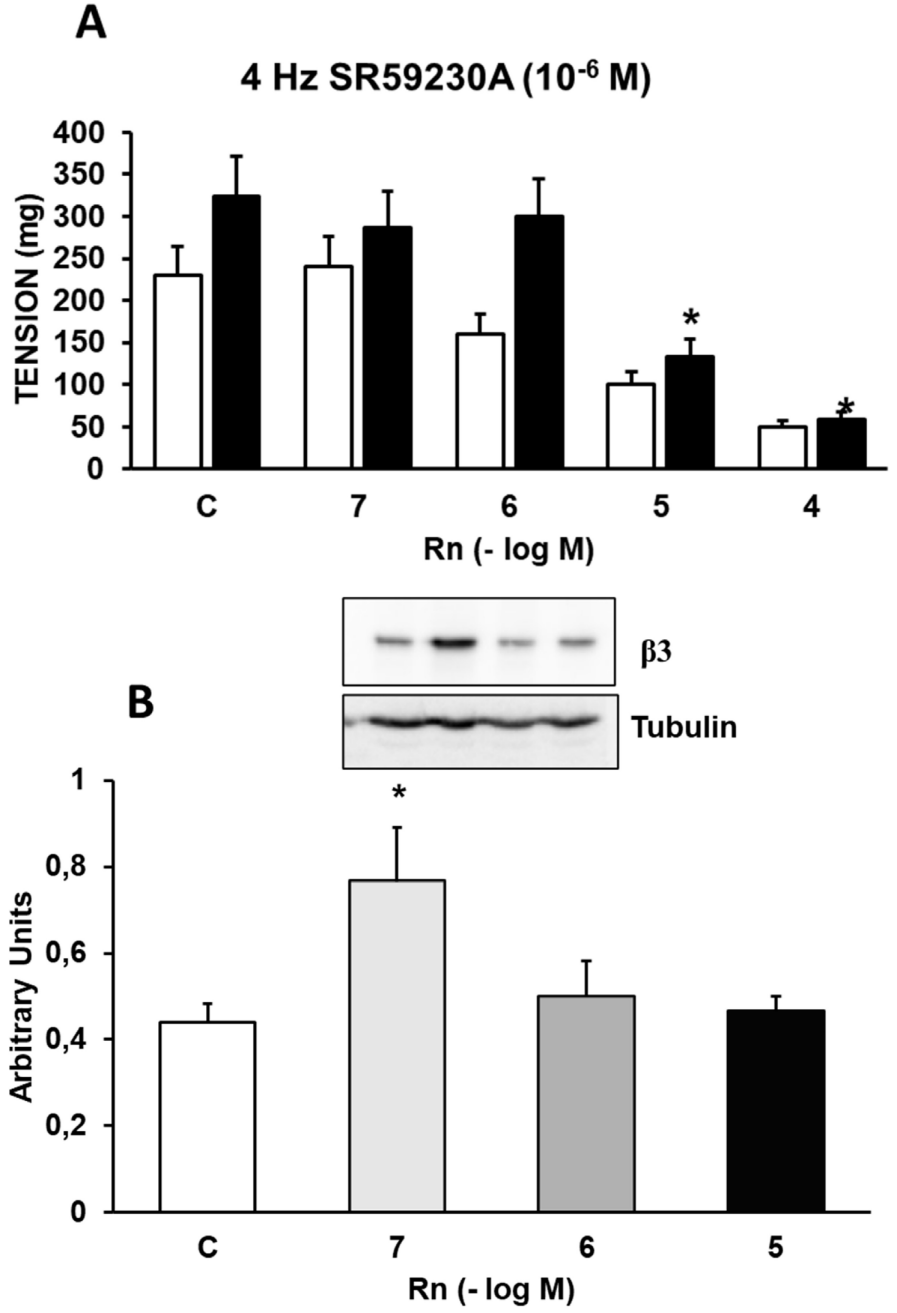
**(A)** Contractile effects of EFS at 4 Hz on rabbit aorta in the absence (control, n = 8) and in the presence of Rn (10^-7^-10^-4^ M) and without SR59230A (□) (n = 8) or previously incubated with SBR59230A (■) (10^-6^ M, n = 9). Values are means ± SEM shown by vertical bars. *p < 0.05 *vs*. control with SR59230A. **(B)** Protein expression of β3 adrenergic receptors by Western-blot. A representative immunoblot is shown in each panel. Data are the mean ± SD of five independent experiments. *p < 0.05 *vs.* control. # < 0,05 *vs*. control sin SR59230A.

Incubation with SR59230A (10⁻⁶ M), a β₃-adrenergic receptor antagonist, increased the contractile response induced by EFS by 29% (Figure 6A) compared to control values (Figure 2A). Rn (10⁻⁷ and 10⁻⁶ M) did not induce changes in the contractile response to EFS. On the other hand, Rn (10⁻⁵ and 10⁻⁴ M) reduced the contractile responses similarly to aortic segments not incubated with SR59230A (Figure 6A). Additionally, Rn induced a very significant increase in β₃ receptor expression only at the concentration of 10⁻⁷ M (Figure 6B). These data suggest that β₃ receptors may be involved in the relaxing responses elicited by Rn.

## Discussion

In our study, we analyzed the effects of sympathetic nervous stimulation on the rabbit aorta by using electrical field stimuli (EFS) capable of causing the release of specific neurotransmitters from the sympathetic nervous system. The response was abolished in the presence of tetrodotoxin, guanethidine, or prazosin.

Rn (10⁻⁶-10⁻⁴ M) inhibited the contractile response to adrenergic nerve stimulation at all stimulation frequencies used in a concentration-dependent manner. In the presence of prazosin, an α₁-adrenergic receptor antagonist, the contractile response to EFS was reduced, while the relaxation induced by Rn began at lower concentrations of Rn (10⁻⁷-10⁻⁴ M). Similarly, Rn decreased the protein expression of α₁-adrenergic receptors.

Yohimbine, an α₂-adrenergic receptor antagonist, like prazosin, reduced the vasoconstrictor response induced by EFS and shifted the vascular relaxation induced by Rn such that this relaxation started at lower concentrations of Rn (10⁻⁷-10⁻⁴ M). Rn decreased the protein expression of α_2_-adrenergic receptors.

Incubation with butaxamine, a β₂-adrenergic receptor antagonist, increased the contractile response to EFS and decreased the relaxation induced by Rn at concentrations of 10⁻⁷ and 10⁻⁶ M. Rn significantly increased the protein expression of β₂-adrenergic receptors only at the concentration of 10⁻⁷ M.

The β₃-adrenergic receptor inhibitor SR59230A potentiated the vasoconstriction induced by EFS and decreased the relaxation induced by Rn at concentrations of 10⁻⁷ and 10⁻⁶ M. Rn significantly increased the protein expression of β₃-adrenergic receptors only at the concentration of 10⁻⁷ M.

Vascular responses to adrenergic nerve stimulation depend on the interaction of noradrenaline with adrenergic receptors located in the vascular wall. In general, the response is vasoconstrictive in both animal 10 and human 20, 21 vessels. This vasoconstrictive response is typically mediated by the binding of noradrenaline to α_1_-adrenergic receptors located on the membrane of vascular smooth muscle fibers 20. In some cases, α_2_-adrenergic receptors 22 also participate, facilitating this vasoconstrictive response. However, other adrenergic receptors such as β_2_ 23 or β_3_ 24 produce a vasodilatory effect when NA binds to them. β_2_ receptors are normally present in vascular smooth muscle, whereas β_3_ receptors are located in vascular endothelial cells. Therefore, the final response to adrenergic nerve stimulation results from the sum of the partial responses generated by each of the receptors. In our study, we observed that Rn inhibits the contractile response to adrenergic nerve stimulation, with its effect beginning at a concentration of 10⁻⁶ M. In the presence of prazosin, an α_1_-adrenergic receptor antagonist, Rn continues to block the contractile response, but this inhibition occurs at lower Rn concentrations (10⁻⁷ M). This finding, together with the fact that Rn inhibits α_1_-adrenergic receptor expression at all concentrations tested, suggests that Rn exerts a clear antagonistic effect on these receptors. These results are consistent with previous studies that proposed an antagonistic effect of Rn on α_1_-adrenergic receptors 25, 19.

As previously mentioned, α_2_-adrenergic receptors also generate a vasoconstrictive response when NA binds to them. In our study, we observed that in the presence of yohimbine, an α_2_-adrenergic receptor antagonist, Rn continued to inhibit the contractile response starting at a concentration of 10⁻⁷ M, like what was observed with prazosin incubation. Likewise, Rn inhibited the protein expression of α_2_-adrenergic receptors at all concentrations tested. Therefore, these results suggest that Rn may also exert an antagonistic effect on α_2_-adrenergic receptors. This antagonistic effect of Rn on α_2_-adrenergic receptors has not been previously described. Regarding β_2_ receptors, our results show that in the presence of butaxamine, an inhibitor of these receptors, the vasoconstrictor response to adrenergic stimulation was greater than the control, and the vasodilatory effect of Rn was abolished at both 10⁻⁷ and 10⁻⁶ M concentrations. Rn increased the expression of β_2_-adrenergic receptors, but only at the 10⁻⁷ M concentration. Therefore, Rn appears to exert a β_2_-agonist effect at the lowest concentration tested. Interactions between Rn and β_2_-adrenergic receptors have been previously reported 26. It has also been reported that ranolazine may exert a weak antagonistic effect on β_2_-adrenergic receptors 27, whereas other studies have found no evidence of interactions between Rn and β_2_ receptors 28. Furthermore, it has been suggested that Rn may act as a weak agonist of β_2_ and β_3_ receptors 29. β_3_-adrenergic receptors induce endothelium-dependent vasodilation. Their activation stimulates endothelial nitric oxide synthase (eNOS) via the PI3K/Akt pathway, increasing the production of nitric oxide (NO) 24, 30. Likewise, β_3_ receptors promote the release of endothelium-derived hyperpolarizing factor (EDHF) 31, generating a protective pathway against endothelial dysfunction. β_3_ receptors act as a counterregulatory mechanism opposing α_1_-adrenergic receptor-mediated vasoconstriction under conditions of high sympathetic activity, thereby limiting excessive increases in peripheral vascular resistance. This function is particularly relevant in pathological conditions such as hypertension, heart failure, and metabolic syndrome 32, and in facilitating endothelial function in patients with hyperglycemia 33. On the other hand, activation of β_3_ receptors can induce the controlled production of reactive oxygen species (ROS), which act as second messengers for eNOS activation. This redox-functional coupling distinguishes β_3_ receptors from other β subtypes, particularly because of their endothelial signaling profile 34. In addition, β3 receptors play a role in protecting against oxidative stress and endothelial inflammation, which is important for preventing vascular dysfunction and the development of cardiovascular diseases 35. Activation of β_3_-adrenergic receptors has been associated with the facilitation of neurocognitive processes 36. SR59230A, a β_3_ antagonist, blocks responses mediated by these receptors, including lipolysis, thermogenesis, and endothelium-dependent vasodilation induced by β_3_ agonists or catecholamines 37, 38. This effect is associated with reduced activation of the PI3K/Akt/eNOS pathway, leading to decreased activating phosphorylation of eNOS (Ser¹¹⁷⁷) and reduced NO bioavailability 24, 32. Blockade of β_3_ receptors with SR59230A abolishes their counterregulatory role against α_1_-adrenergic vasoconstriction. Therefore, an enhanced vasoconstrictor response to different adrenergic agents is observed, accompanied by an increase in basal vascular tone. This effect has been described in resistance vessels and may be key in situations of sympathetic hyperactivity 39. Several studies suggest that SR59230A promotes a specific type of endothelial dysfunction characterized by reduced NO availability and an altered redox balance, such that blockade of β_3_ signaling leads to a predominance of deleterious ROS and functional uncoupling of eNOS 34.

In our study, Rn at a concentration of 10⁻⁷ M increases the expression of β_2_ and β_3_ receptors. However, at higher concentrations (10⁻⁶ and 10⁻⁵ M), Rn does not induce any change in the expression of these receptors. There may be several explanations for the mechanisms underlying this response. One possibility is that Rn, at low doses, activates the protein kinase A (PKA) signaling pathway 40, which could lead to an increase in β_3_ receptor expression. In contrast, at higher doses, it could activate the protein kinase C (PKC) signaling pathway, which may inhibit the expression of β_3_ receptors 41, 42. It has been shown that β₂-adrenergic receptors undergo desensitization following sustained activation, which involves PKA- and GRK-dependent phosphorylation, recruitment of β-arrestins, and subsequent receptor internalization, ultimately reducing their expression on the cell surface 29, 43. Another possibility is that at low doses, Rn could bind to β_3_ receptors, whereas at higher doses it might interact with other adrenergic receptors, such as α_1_, which could inhibit the expression of β_2_ or β_3_ receptors 25. Furthermore, it has been reported that ranolazine can exert an antagonistic effect on β_2_-adrenergic receptors at doses corresponding to therapeutic concentrations of Rn (10⁻⁶-10⁻⁵ M). There are no studies on a potential antagonistic effect of Rn at these concentrations on β_3_ receptors, but this could be a possible mechanism 27. This may be due to unequal selectivity of ranolazine for different adrenergic receptors 44.

These interactions with adrenergic receptors could explain various effects attributed to Rn. Among these, the cardiovascular effects in the treatment of coronary ischemia 2 and different cardiac arrhythmias 45, 46, such as atrial fibrillation, are particularly noteworthy. Rn improves coronary endothelial dysfunction 47, reduces levels of asymmetric dimethylarginine and C-reactive protein, and increases endothelial release of vasodilator mediators such as nitric oxide 48. Furthermore, it improves both systolic and diastolic heart failure 49, prevents oxidative stress 50, and reduces hypertrophic cardiomyopathy 51. Similarly, the interactions of Rn with adrenergic receptors could be involved in the drug's metabolic effects. In patients with type 2 diabetes, Rn decreases blood glucose and glycated hemoglobin (HbA1c) levels 52, 53. It has also been observed that Rn may reduce glucagon release through the opening of sodium channels, thereby improving pre-prandial and postprandial glucose levels 54. It has also been demonstrated that Rn improves cognitive processes in these patients 7. In addition, Rn exhibits anticonvulsant properties in epileptic seizures 55, has shown efficacy in the treatment of neuropathic pain 13, and exerts neuroprotective effects in different types of dementia 15. However, the mechanisms underlying these effects have not yet been fully elucidated. It is possible that different adrenergic receptors are involved in these processes. In recent years, evidence has emerged suggesting that Rn may reduce tissue insulin resistance by increasing sensitivity to the hormone. In a previous study conducted in our laboratory, we demonstrated that Rn improves vascular sensitivity to insulin. In this effect, α_1_-adrenergic antagonism induced by Rn plays an important role 10. It would be interesting to further investigate the potential involvement of α- or β-adrenergic receptors in the mechanisms facilitating tissue insulin sensitivity or in other effects attributed to Rn.

## Conclusions

According to the data obtained in our study, Rn inhibits vasoconstrictor responses to adrenergic nerve stimulation in the rabbit aorta. This inhibition is concentration-dependent, and it may be due to an antagonistic effect of Rn on α_1_- and α_2_-adrenergic receptors, as well as an enhancing effect on β_2_- and β_3_-adrenergic receptors. Furthermore, Rn, at all concentrations tested, decreases the expression of the vasoconstrictor α_1_- and α_2_-receptors and increases the expression of the vasodilatory β_2_- and β_3_-receptors, but only at the 10⁻⁷ M concentration, which corresponds to a dose lower than that used in clinical practice. This finding may represent a point of clinical interest, as it reflects a potential therapeutic effect.

## Abbreviations

Rn: ranolazine
EFS: electrical field stimulation
PRZ: prazosin
TTX: tetrodotoxin
GN: Guanethidine
